# Investigation of the Effect of Zebularine in Comparison to and in Combination with Trichostatin A on *p21Cip1/Waf1/Sdi1*,* p27Kip1*, *p57Kip2*, DNA Methyltransferases and Histone Deacetylases in Colon Cancer LS 180 Cell Line

**DOI:** 10.31557/APJCP.2020.21.6.1819

**Published:** 2020-06

**Authors:** Masumeh Sanaei, Fraidoon Kavoosi

**Affiliations:** *Research Center for Non-communicable Diseases, Jahrom University of Medical Sciences, Jahrom, Iran. *

**Keywords:** Zebularine, trichostatin, TSGs, colon cancer

## Abstract

**Background:**

The heart of the cell cycle regulatory machine is a group of enzymes named cyclin-dependent kinases (Cdks). The active form of these enzymes includes a kinase and its partner, a cyclin. The regulation of cyclin-Cdk complexes is provided by Cdk inhibitors (CKIs) such as Cip/Kip family comprising p21Cip1/Waf1/Sdi1, p27Kip1, and p57Kip2. The hypermethylation and deacetylation of *Cip/Kip* gene family seem to be frequent in numerous cancers. It has been indicated that increased expression of DNMTs and HDACs contributes to cancer induction. Previously, we reported the effect of DNA demethylating agents and histone deacetylase inhibitors on histone deacetylase 1, DNA methyltransferase 1, and CIP/KIP family in colon cancer. The current study was designed to evaluate the effect of zebularine in comparison to and in combination with trichostatin A (TSA) on *p21Cip1/Waf1/Sdi1*, *p27Kip1*,* p57Kip2*, DNA methyltransferases (DNMT1, 3a and 3b) and histone deacetylases (*HDAC1*, *2*, and *3*) genes expression, cell growth inhibition and apoptosis induction in colon cancer LS 180 cell line.

**Materials and Methods:**

The colon cancer LS 180 cell line was cultured and treated with zebularine and TSA. To determine cell viability, apoptosis, and the relative expression level of the genes, MTT assay, cell apoptosis assay, and qRT-PCR were done respectively.

**Results:**

Both compounds significantly inhibited cell growth, and induced apoptosis. Furthermore, both compounds increased *p21Cip1/Waf1/Sdi1*,* p27Kip1*, and *p57Kip2* significantly. Additionally, zebularine and* TSA* decreased* DNMTs* and *HDACs *gene expression respectively. Conclusion: The zebularine and TSA can reactivate the CIP/KIP family through inhibition of DNMTs and HDACs genes activity.

## Introduction

The mammalian cell cycle refers to the determined intervals during which the cells prepare to duplicate their genome equally between two new daughter cells. This cycle is divided into four consecutive phases (G1, G2, S, and M phase) and comprises a series of regulated events controlling mitosis and proliferation to produce two daughter cells from a single cell, cell division is tightly controlled to ensure precise genome duplication (Matson et al., 2017). During the G1 phase, the cell accumulates necessary metabolites for DNA replication. Subsequently, the cell enters the S phase during which DNA is replicated. G2 phase is essential to ensure accurate DNA replication and finally, the cell is divided during M phase (Qie et al., 2016). The heart of the cell cycle regulatory machine is a group of enzymes named cyclin-dependent kinases (Cdks). The active form of these enzymes includes a kinase and its partner, a cyclin, together form a complex to derive cell cycle progression. The change in the kinase or cyclin components drives the cell from one phase to another phase of the cycle. A sequence of kinase subunits, *Cdk4*, *Cdk6*, *Cdk2*, and *Cdk2*, along with a sequence of cyclins, cyclin D, E, A, and B, are expressed during the cell progresses from G1 phase to mitosis (Park et al., 2008). Furthermore, the regulation of cyclin-Cdk complexes is provided by Cdk inhibitors (CKIs). The *CKI* gene families are divided into two groups based on structure and evolutionary origins consisting* INK4* gene family which encodes p16INK4a, p15INK4b, p18INK4c, and p19INK4d and Cip/Kip family comprising p21Cip1/Waf1/Sdi1, p27Kip1, and p57Kip2 (Besson et al., 2008). The hypermethylation of the* INK4 *gene family (Wong et al., 2000; Chim et al., 2001) and *Cip/Kip* gene family (Yoshino et al., 2007; Zohny et al., 2017) seems to be frequent in numerous cancers. Another mechanism by which chromatin is inactivated is histone deacetylation reported in the hundreds of various cancers (Fang et al., 2002; Kikuchi et al., 2002). The Cip/Kip family could be inactivated by this pathway as reported in MCF-7 breast cancer (Varshochi et al., 2005), pancreatic cancer (Jiao et al., 2014), colon cancer (Chen et al., 2009), thyroid cancer (Weinlander et al., 2014), and gastric cancer (Sun et al., 2014). Primary regulators of *INK4* and* Cip/Kip* family genes include DNA histone deacetylases (HDACs) and DNA methyltransferases (DNMTs). It has been indicated that increased expression of* DNMTs* and *HDACs* contribute to cancer induction through methylation- and deacetylation-mediated gene inactivation in various cancers (Patra et al., 2001). The over-expression of DNMTs (DNMT1, 3A, and 3B) has been shown in uterine cancer (Li et al., 2003), breast cancer (Girault et al., 2003), hepatocellular carcinoma (HCC) (Nagai et al., 2003), colorectal and stomach cancer (Kanai et al., 2001). Furthermore, high HDACs (HDACs 1, 2 and 3) expression levels are found in breast cancer (Müller et al., 2013), ovarian cancer (Khabele., 2014), bladder cancer (Poyet et al., 2014), and renal cancer (Fritzsche et al., 2008). DNA methyltransferase inhibitors (*DNMTIs*) can reactivate hypermethylated genes. It has been shown that DNMT-inhibiting cytosine nucleoside analogs, decitabine, azacitidine, and zebularine, have a significant effect on several cancers such as myeloid leukemia (AML) (Flotho et al., 2009), breast cancer (Chen et al., 2012), gastric cancer (Tan et al., 2013), colorectal cancer (His et al., 2005), endometrial cancer (Cui et al., 2010), lung cancer (Luszczek et al., 2010), colorectal cancer (Xiong et al., 2009), and prostate cancer (Walton et al., 2008). The HDACIs (carboxylic acids, hydroxamic acids, benzamides, and cyclic tetrapeptides) are other compounds that can restore silenced tumor suppressor genes (*TSGs*). They inhibit Class I (HDAC1, 2, 3, and 8) and class II HDACs (HDAC4, 5, 6, 7, 9, and 10) in various cancers including colon cancer, lung cancer, breast cancer, gastric cancer, and pancreatic cancer cells (Chueh et al., 2015). Previously, we reported the effect of DNA demethylating agent 5-aza-2′-deoxycytidine (decitabine, 5AZA-CdR) and histone deacetylase inhibitors valproic acid (VPA) and trichostatin A (TSA) on histone deacetylase 1, DNA methyltransferase 1, and Cip/Kip family (*p21*, *p27*, and *p57*) genes expression, cell growth inhibition, and apoptosis induction in colon cancer SW480 cell line (Sanaei et al., 2018). The current study was designed to evaluate the effect of zebularine in comparison to and in combination with trichostatin A on p21Cip1/Waf1/Sdi1, p27Kip1, p57Kip2, DNA methyltransferases (*DNMT1*, *3a*, and *3b*) and histone deacetylases (*HDAC1*, *2*, and *3*) genes expression, cell growth inhibition and apoptosis induction in colon cancer LS 180 cell line. 

## Materials and Methods

*Materials*

The human colon cancer LS 180 cell line was provided from the National Cell Bank of Iran-Pasteur Institute and maintained in Dulbecco’s modified Eagle’s medium (DMEM) supplemented with fetal bovine serum 10% and antibiotics in a humidified atmosphere of 5% CO_2 _in air at 37^o^C. Zebularine, TSA, and 3 (4,5 dimethyl 2 thiazolyl) 2, 5 diphenyl 2H tetrazolium bromide (MTT) were obtained from Sigma–Aldrich (Sigma–Aldrich, Louis, MI, USA) and the Annexin V and also propidium iodide (PI) apoptosis kit from Life Technologies. Total RNA extraction kit (TRIZOL reagent) and Real-time polymerase chain reaction (PCR) kits (qPCR MasterMix Plus for SYBR Green I dNTP) were purchased from Applied Biosystems Inc. (Foster, CA, USA) and Dimethyl sulfoxide (DMSO) from Merck Co. (Darmstadt, Germany). 

*Cell culture and cell viability*

To determine cell viability, colon cancer LS 180 cell line was cultured in DMEM supplemented with 10% FBS and antibiotics and incubated overnight and then seeded into 96-well plates (4 × 10^5^ cells per well). Next day, the culture medium was replaced by experimental medium containing various concentration of zebularine (0, 5, 10, 25, 50 and 100 μM) and TSA (0, 1, 2.5, 5, 10, and 25 μM) for 24 and 48 h, except control groups which treated with DMSO at a concentration of 0.05 %. Subsequently, the colon cancer LS 180 cell viability was assessed by MTT assay according to Standard protocols. First, the MTT solution was added for 4 h at 37^o^C and then the MTT solution was changed with DMSO. To dissolve all of the crystals, the solution was shaken for 10 min. Finally, the optical density was detected by a microplate reader at a wavelength of 570 nM. Each experiment was repeated three times (triplicates).

*Cell apoptosis assay*

For apoptotic cell investigation, the colon cancer LS 180 cell line were cultured at a density of 4 × 10^5^ cells/well and treated with zebularine (50 μM) and TSA (2.5 μM), individually and combined, for 24 and 48 h. After treatment, the cells were harvested by trypsinization, washed twice in PBS and resuspended in Binding buffer (1x). Next, annexin V-FITC and PI were added to obtain the apoptotic cells. After incubation at room temperature, 15 min in the dark, the apoptotic cells were counted by FACScan flow cytometry (Becton Dickinson, Heidelberg, Germany).

*Real-time Quantitative Reverse Transcription Polymerase*

*Chain Reaction (qRT-PCR)*

The qRT-PCR was done to investigate the relative expression level of p21Cip1/Waf1/Sdi1, p27Kip1, p57Kip2, DNA methyltransferases (DNMT1, 3a, and 3b) and histone deacetylases (*HDAC1*, *2*, and *3*) genes. The colon cancer LS 180 cell line was treated with zebularine (50 μM) and TSA (2.5 μM), individually and combined, for 24 and 48 h, based on IC5o values, and total RNA was extracted using the RNeasy kit (Qiagen, Valencia, CA) according to the manufacturer protocol and treated by RNasefree DNase (Qiagen). Other processes were done as we did previously (Sanaei et al., 2018). The primer sequences of the genes are indicated in Table 1. GAPDH was used as an endogenous control. Data were analyzed using the comparative Ct (ΔΔct) method.

## Results

*Result of cell viability by the MTT assay*

**Figure 1:**
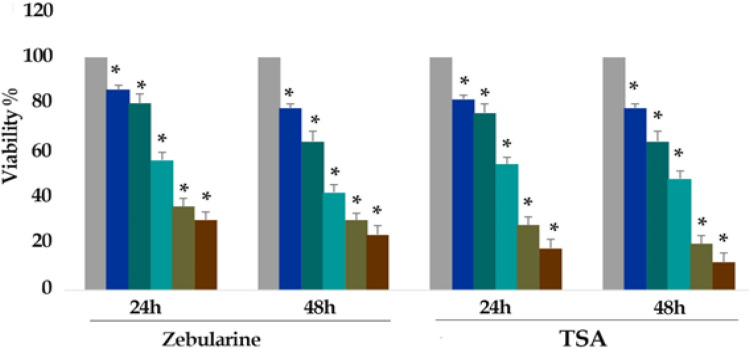
In vitro Effects of Zebularine (0, 5, 10, 25, 50 and 100 μM) and TSA (0, 1, 2.5, 5, 10, and 25 μM) on LS 180 Cells Viability Determined by MTT Assay at 24 and 48 h. As shown in figure 1, from right to the left, the first column of each group belongs to the control group. Values are means of three experiments in triplicate. Asterisks (*) demonstrate significant differences between treated and untreated control groups

The viability of the LS 180 cell line was assessed by MTT assay. As mentioned above, the cells were treated with zebularine (0, 5, 10, 25, 50 and 100 μM) and TSA (0, 1, 2.5, 5, 10, and 25 μM) for 24 and 48 h and the cell viability was determined based on the activity of cellular enzymes to reduce the tetrazolium salt MTT resulting in a dark-blue formazan. To determine the number of viable cells, the product was dissolved in DMSO. The result of the assay indicated that both compounds significant cell growth inhibition with all used concentrations at different periods as shown in Figure 1 (P< 0.001).

The IC50 values were determined with approximately 50 and 2.5 μM for zebularine and TSA respectively. 

*Result of cell apoptosis assay*

**Figure 2:**
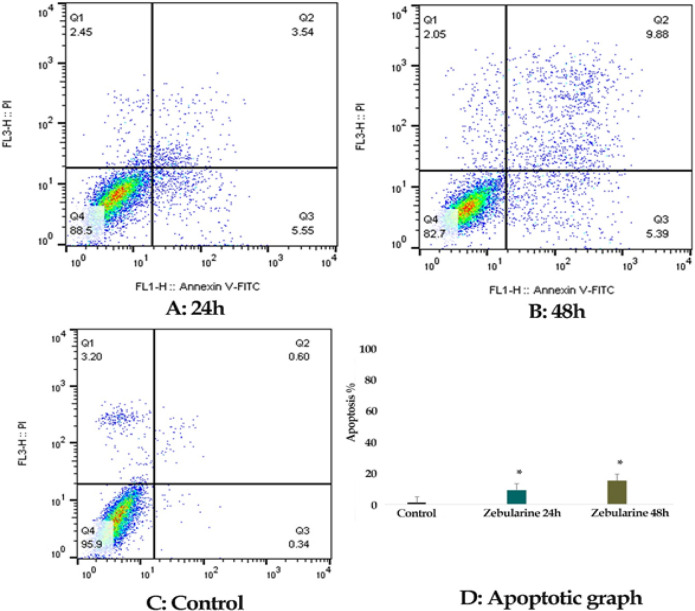
The Apoptotic Effect of Zebularine (50 μM) on LS 180 Cell versus Control Groups at Different Periods (24 and 48h). The cells were treated with this agent for 24 and 48h and then the apoptotic effect was evaluated by flow cytometric analysis. Results were obtained from three independent experiments and were expressed as mean ± standard error of the mean

**Figure 3:**
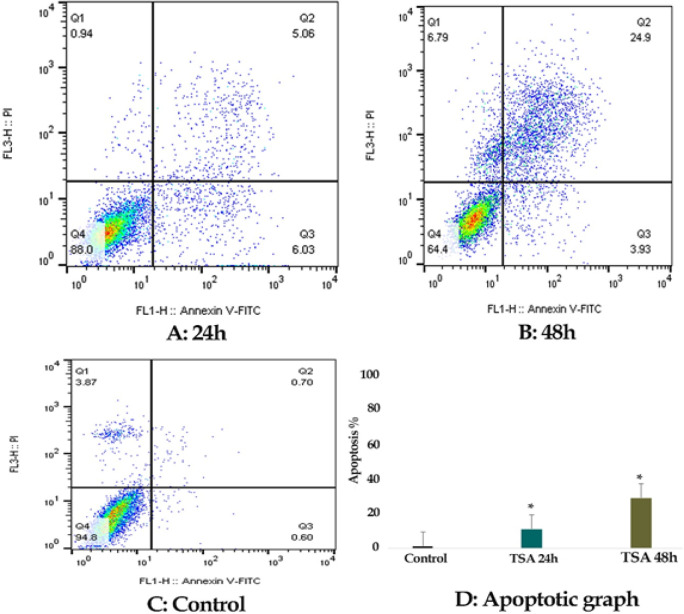
The Apoptotic Effect of TSA (2.5 μM) on LS 180 Cell versus Control Groups at Different Periods (24 and 48h). The cells were treated with this agent for 24 and 48h and then the apoptotic effect was evaluated by flow cytometric analysis. Results were obtained from three independent experiments and were expressed as mean ± standard error of the mean

**Figure 4:**
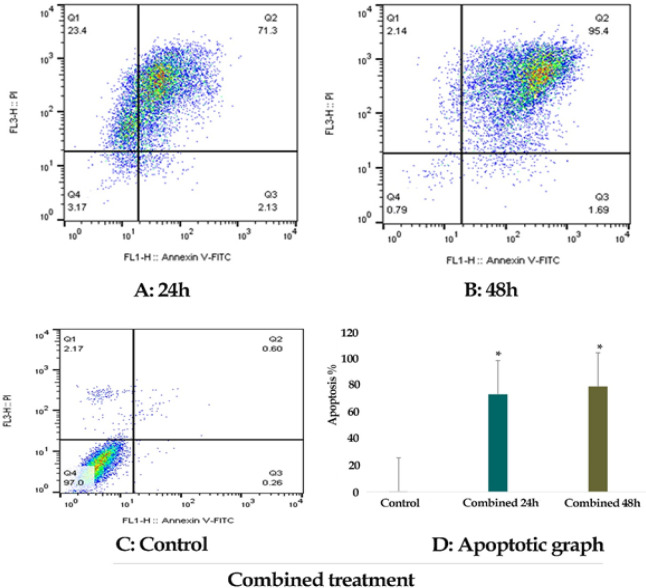
The Apoptotic Effect of Zebularine (50 μM) in Combination with TSA (2.5 μM) for 24 and 48 h on LS 180 Cell versus Control Groups at Different Periods (24 and 48h). The cells were treated with this agent for 24 and 48h and then the apoptotic effect was evaluated by flow cytometric analysis. Results were obtained from three independent experiments and were expressed as mean ± standard error of the mean

**Figure 5:**
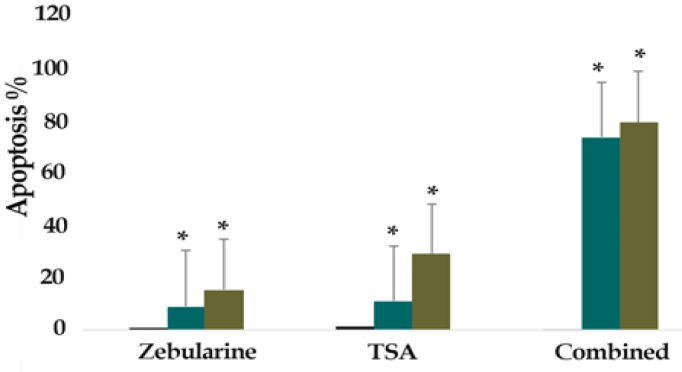
The Comparative Apoptotic Effects of Zebularine (50 μM) in Comparison to and in Combination with TSA (2.5 μM) on LS 180 cells. Asterisks (*) indicate significant differences between the treated and untreated control groups. As demonstrated above, TSA had a more significant apoptotic effect on LCL-PI 11 cells in comparison to zebularine. The combined treatment had a maximum effect on apoptosis

The percentage of LS 180 apoptotic cells was evaluated by staining with annexin V-FITC AND PI. The cells were treated with zebularine (50 μM) and TSA (2.5 μM) individually and combined for 24 and 48 h and then stained using annexin-V-(FITC) and PI. The result indicated that both compounds induced significant apoptosis as alone and combined, Figures 2-4. Further, TSA had a more significant effect in comparison to zebularine. Maximal apoptotic effect was observed with combined treatment after 48 h and minimal apoptosis was seen with zebularine at 24 h, Figure 5. The percentage of apoptosis has been shown in Table 2.

*Result of determination of genes expression*

**Figure 6:**
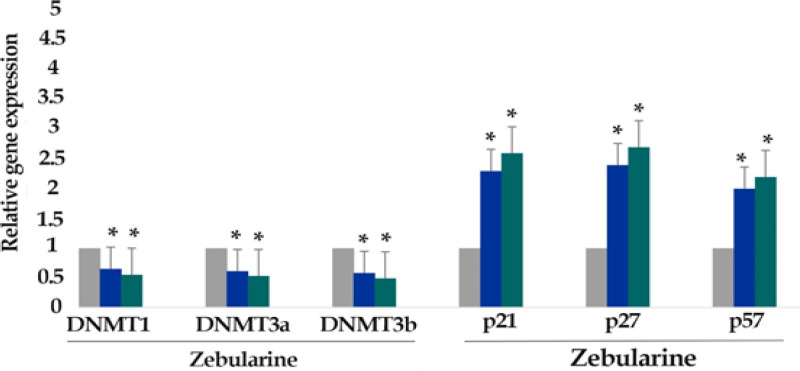
The Relative Expression Level of DNA Methyltransferases *(DNMT1,* 3a, and 3b), *p21Cip1/Waf1/Sdi1, p27Kip1*, and *p57Kip2* in the LS 180 cell line treated with zebularine (50 μM) versus untreated control groups at different periods (24 and 48h). The first column of each group belongs to the untreated control group and the others belong to the treated cells with zebularine. Asterisks (*) indicate significant differences between the treated and untreated groups

**Figure 7:**
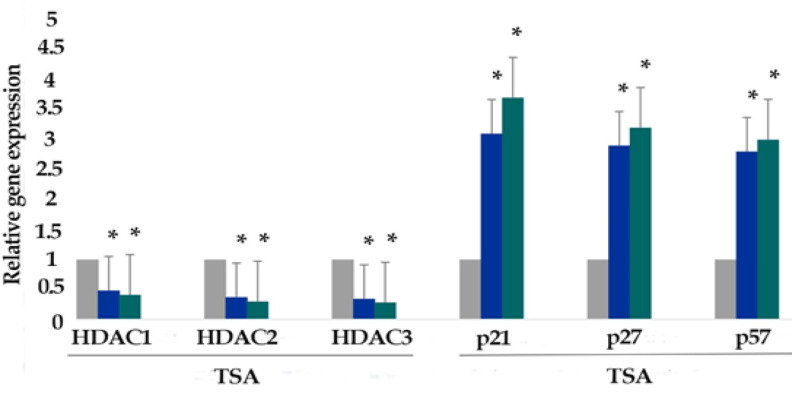
The Relative Expression Level of Histone Deacetylases (*HDAC1*, 2, and 3), *p21Cip1/Waf1/Sdi1, p27Kip1, *and *p57Kip2* in the LS 180 cell line treated with TSA (2.5 μM) versus untreated control groups at different periods (24 and 48h). The first column of each group belongs to the untreated control group and the others belong to the treated cells with zebularine. Asterisks (*) indicate significant differences between the treated and untreated groups

**Figure 8:**
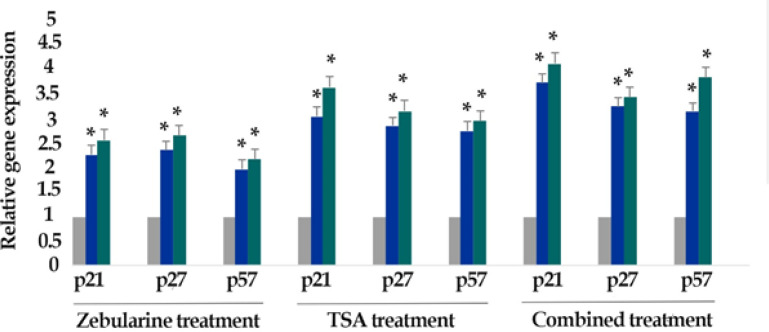
The Relative Expression Level of *p21Cip1/Waf1/Sdi1, p27Kip1,* and *p57Kip2* in the LS 180 cell line treated with combined compounds versus untreated control groups at different periods (24 and 48h). The first column of each group belongs to the untreated control group and the others belong to the treated cells with zebularine in combination with TSA. Asterisks (*) indicate significant differences between the treated and untreated groups

The effect of zebularine (50 μM) and TSA (2.5 μM), alone and combined, on the relative expression level of p21Cip1/Waf1/Sdi1, p27Kip1, p57Kip2, DNA methyltransferases (DNMT1, 3a, and 3b) and histone deacetylases (*HDAC1*, *2*, and *3*) genes was determined by quantitative real-time RT-PCR analysis. The finding indicated that zebularine decreased DNA methyltransferases (DNMT1, 3a and 3b), decreased DNA methyltransferases (DNMT1, 3a and 3b) and histone deacetylases (HDAC1, 2, and 3), and both compounds increased *p21Cip1/Waf1/Sdi1, p27Kip1, p57Kip2* genes expression significantly in LS 180 cell line after 24 and 48 h, Figure 6 and Figure 7. Additionally, TSA had a more significant effect on the up-regulation of p21Cip1/Waf1/Sdi1, p27Kip1, and p57Kip2 in comparison to zebularine. Further, the maximum expression of *p21Cip1/Waf1/Sdi1*, *p27Kip1*, and *p57Kip2* genes was observed with combined treatment as demonstrated in Figure 8. The relative expression level of the genes has been indicated in Table 3 and 4.

**Table 1:** The Primer Sequences of *p21Cip1/Waf1/Sdi1, p27Kip1, p57Kip2,* DNA Methyltransferases (*DNMT1*, 3a, and 3b) and Histone Deacetylases (*HDAC1*, 2, and 3) Genes

Primer name	Primer sequences (5' to 3')	References
DNMT1 Forward	GAG GAA GCT GCT AAG GAC TAG TTC	Sanaei et al., 2018
DNMT1 Reverse	ACT CCA CAA TTT GAT CAC TAA ATC	Sanaei et al., 2018
DNMT3a Forward	GGA GGC TGA GAA GAA AGC CAA GGT	Sanaei et al., 2018
DNMT3a Reverse	TTT GCC GTC TCC GAA CCA CAT GAC	Sanaei et al., 2018
DNMT3b Forward	TAC ACA GAC GTG TCC AAC ATG GGC	Sanaei et al., 2018
DNMT3b Reverse	GGA TGC CTT CAG GAA TCA CAC CTC	Sanaei et al., 2018
P21 Forward	AGG CGC CAT GTC AGA ACC GGC TGG	Sanaei et al., 2019
P21 Reverse	GGA AGG TAG AGC TTG GGC AGG C	Sanaei et al., 2019
P 27 Forward	ATG TCA AAC GTG CGA GTG TCT AAC	Sanaei et al., 2019
P 27 Reverse	TTA CGT TTG ACG TCT TCT GAG GCC A	Sanaei et al., 2019
P 57 Forward	GCGGCGATCAAGAAGCTGTC	Sanaei et al., 2019
P 57 Reverse	CCGGTTGCTGCTACATGAAC	Sanaei et al., 2019
GAPDH Forward	TCCCATCACCATCTTCCA	Sanaei et al., 2019
GAPDH Reverse	CATCACGCCACAGTTTCC	Sanaei et al., 2019
HDAC1 Forward	AACCTGCCTATGCTGATGCT	Jin et al., 2008
HDAC1 Reverse	CAGGCAATTCGTTTGTCAGA	Jin et al., 2008
HDAC2 Forward	GGGAATACTTTCCTGGCACA	Jin et al., 2008
HDAC2 Reverse	ACGGATTGTGTAGCCACCTC	Jin et al., 2008
HDAC3 Forward	TGGCTTCTGCTATGTCAACG	Jin et al., 2008
HDAC3 Reverse	GCACGTGGGTTGGTAGAAGT	Jin et al., 2008

**Table 2:** Percentage of Apoptosis in the Groups Treated with Zebularine and TSA, as alone and Combined, at Different Periods

Drug	Dose/ μM	Duration/ h	Apoptosis %	*P*-value
Zebularine	25	24	9.09	P < 0.001
	25	48	15.27	P < 0.001
TSA	5	24	11.09	P < 0.001
	5	48	28.83	P < 0.001
Zebularine/TSA	25/5	24	73.43	P < 0.001
	25/5	48	79.09	P < 0.001

**Table 3.** The Relative Expression Level of *p21Cip1/Waf1/Sdi1, p27Kip1, p57Kip2, DNMTs, HDAC1, HDAC2*, and *HDAC3* Genes

Cell line	Gene	Drug	Dose (μM)	Duration (h)	Expression	*P*-value
LS 180	*DNMT1*	Zebularine	2.5 μM	24	0.65	0.001
LS 180	*DNMT1*	Zebularine	25 μM	48	0.55	0.001
LS 180	*DNMT3a*	Zebularine	25 μM	24	0.61	0.001
LS 180	*DNMT3a*	Zebularine	25 μM	48	0.53	0.001
LS 180	*DNMT3b*	Zebularine	25 μM	24	0.58	0.001
LS 180	*DNMT3b*	Zebularine	25 μM	48	0.49	0.01
LS 180	*P21*	Zebularine	25 μM	24	2.3	0.004
LS 180	*P21*	Zebularine	25 μM	48	2.6	0.001
LS 180	*P27*	Zebularine	25 μM	24	2.4	0.001
LS 180	*P27*	Zebularine	25 μM	48	2.7	0.001
LS 180	*P57*	Zebularine	25 μM	24	2	0.001
LS 180	*P57*	Zebularine	25 μM	48	2.2	0.001
LS 180	*HDAC1*	Trichostatin A	5 μM	24	0.48	0.001
LS 180	*HDAC1*	Trichostatin A	5 μM	48	0.41	0.004
LS 180	*HDAC2*	Trichostatin A	5 μM	24	0.37	0.002
LS 180	*HDAC2*	Trichostatin A	5 μM	48	0.3	0.01
LS 180	*HDAC3*	Trichostatin A	5 μM	24	0.34	0.007
LS 180	*HDAC3*	Trichostatin A	5 μM	48	0.28	0.001
LS 180	*P21*	Trichostatin A	5 μM	24	3.1	0.001
LS 180	*P21*	Trichostatin A	5 μM	48	3.7	0.001
LS 180	*P27*	Trichostatin A	5 μM	24	2.9	0.001
LS 180	*P27*	Trichostatin A	5 μM	48	3.2	0.001
LS 180	*P57*	Trichostatin A	5 μM	24	2.8	0.001
LS 180	*P57*	Trichostatin A	5 μM	48	3	0.001

**Table 4:** The Relative Expression Level of* p21Cip1/Waf1/Sdi1, p27Kip1, p57Kip2* Genes with Combined Treatment

Cell line	Gene	Drug	Dose (μM)	Duration (h)	Expression	*P*-value
LS 180	*P21*	Zebularine/TSA	25/5 μM	24	3.8	0.001
LS 180	*P21*	Zebularine/TSA	25/5 μM	48	4.2	0.001
LS 180	*P27*	Zebularine/TSA	25/5 μM	24	3.3	0.001
LS 180	*P27*	Zebularine/TSA	25/5 μM	48	3.5	0.001
LS 180	*P57*	Zebularine/TSA	25/5 μM	24	3.2	0.001
LS 180	*P57*	Zebularine/TSA	25/5 μM	48	3.9	0.001

## Discussion

Epigenetic regulation such as DNA methylation and histone modification is the mechanism by which gene is activated or inactivated in the mammalian cells. This mechanism is more specified genetic information and involved in gene repression. Recent studies have identified a variety of regulatory proteins comprising histone-modifying enzymes, DNA methyltransferases, chromatin remodeling factors, and methyl-CpG binding proteins. Abnormalities and changes in the epigenetic states such as DNA hypermethylation and histone deacetylation represent several diseases, especially tumorigenesis. However, promoter hypermethylation and histone deacetylation play a significant role in cancer through transcriptional silencing of TSGs. Meanwhile, the DNA demethylating agents and histone deacetylase inhibitors can induce re-expression of silenced TSGs causing growth arrest and apoptosis (Baylin., 2007; Kelly et al., 2003). In this study, we observe that zebularine and TSA can up-regulate p21Cip1/Waf1/Sdi1, p27Kip1, p57Kip2 and down-regulate DNA methyltransferases (DNMT1, 3a, and 3b) and histone deacetylases *(HDAC1*, *2*, and* 3*) gene expression resulting in cell growth inhibition and apoptosis induction. Further, the effect of TSA was stronger than that of zebularine. Maximal expression of p21Cip1/Waf1/Sdi1, p27Kip1, p57Kip2 and also cell apoptosis was seen with combined treatment. Inconsistent with our result, it has been reported that zebularine can increase the level of p21Cip1/Waf1/Sdi1 in SW48 cells (Flis et al., 2014). The other members of DNMTIs act by a similar pathway. In vitro studies have shown that 5-aza increases p21WAF1 in colon cancer Colo-320 and SW1116 (Fang et al., 2004; Chen et al., 2004). The re-activation of Cip/Kip family by DNMTIs has been shown in several cancers such as myeloma cell line WL2 (Chim et al., 2005), human pancreatic cancer (Wang et al., 2013), and gastric cancer (Pellegrini et al., 2010). In addition to Cip/Kip, zebularine can up-regulate ink4 p15INK4b and p16INK4a in colon cancer Caco-2 cell (Berner et al., 2010), and also p53 in colon cancer (Yang et al., 2013) and other cancer such as HCC (Nakamura et al., 2013). In contrast, some studies have shown that zebularine is not effective on the up-regulation of p21 and p27 in colon cancer HCT15, SW48, and HT-29 colon cancer (Cheng et al., 2004). 

In the current study, we indicated that TSA can up-regulate *p21Cip1/Waf1/Sdi1, p27Kip1, p57Kip2 *genes significantly. As we reported, other studies have been demonstrated that TSA up-regulates p21Cip1/Waf1/Sdi1 in colon cancer SW480 and HT-29 cell lines (Spurling et al., 2008), p27Kip1 in colon cancer HT-29 M6 (Mayo et al., 2007). The same pathway, Cip/Kip up-regulation, has been demonstrated in various cancers including, lung cancer (Platta et al., 2007), HCC Hep3B cells (Svechnikova et al., 2007), and human gastric cancer cell lines, OCUM-8 and MKN-74 (Zhang et al., 2006). We observed that cell apoptosis was increased after combined treatment. Other researchers have demonstrated that apoptosis induction is greatly enhanced in the presence of combined treatment, HDACIs in combination with DNMTIs, in human lung, thoracic, breast, leukemia and colon cancer cell lines (Zhu et al., 2003; Belinsky et al., 2003), and breast cancer (Primeau et al., 2003). After the evaluation of gene expression, we performed a further investigation and found that zebularine and TSA induce this effect through inhibition of DNMTs and HDACs respectively. Such inhibitory effect on DNMTs has been shown in T24 bladder cancer, HCT15, SW48, and HT-29 colon cancer, CFPAC-1 pancreatic cancer, PC3 prostate cancer, CALU-1 lung cancer cells [48], cholangiocarcinoma (CCA) TFK-1 and HuCCT1 cell lines (Nakamura et al., 2015). Additionally, the similar inhibitory effect of TSA on HDAC Class I has been demonstrated in other cancers comprising gastric MKN-7, MKN-28 and Ho-1-N-1, and oral Ho-1-N-1 cell lines (Suzuki et al., 2000). It should be noted that the inhibition of HDAC Class I is not the only mechanism of TSA. It can inhibit other HDACs including class I, II, and IV (Rikiishi., 2011). Summery, we indicated that zebularine and TSA down-regulate DNA methyltransferases (DNMT1, 3a, and 3b) and histone deacetylases (HDAC1, 2, and 3) by which up-regulate *p21Cip1/Waf1/Sdi1, p27Kip1, p57Kip2* gene expression resulting in LS 180 cell growth inhibition and apoptosis induction.

In conclusion, our findings demonstrated that zebularine and TSA can epigenetically down-regulate *DNMTs* and *HDACs *gene expression by which re-activate the *Cip/Kip* family gene in colon cancer LS 180 cell line cells resulting in cell growth inhibition, and apoptosis induction. Thus, this result suggests a dependence of the *p21Cip1/Waf1/Sdi1*, and *p27Kip1* gene silencing through histone deacetylation and DNA hypermethylation by a mechanism that involves the up-regulation of histone deacetylases and DNA methyltransferases. 
